# Using zero-inflated and hurdle regression models to analyze schistosomiasis data of school children in the southern areas of Ghana

**DOI:** 10.1371/journal.pone.0304681

**Published:** 2024-07-12

**Authors:** Kojo Nketia, Dziedzom K. de Souza

**Affiliations:** Department of Parasitology, Noguchi Memorial Institute for Medical Research, University of Ghana, Legon, Ghana; Universite Felix Houphouet-Boigny, SWITZERLAND

## Abstract

**Background:**

Schistosomiasis is a neglected disease prevalent in tropical and sub-tropical areas of the world, especially in Africa. Detecting the presence of the disease is based on the detection of the parasites in the stool or urine of children and adults. In such studies, typically, data collected on schistosomiasis infection includes information on many negative individuals leading to a high zero inflation. Thus, in practice, counts data with excessive zeros are common. However, the purpose of this analysis is to apply statistical models to the count data and evaluate their performance and results.

**Methods:**

This is a secondary analysis of previously collected data. As part of a modelling process, a comparison of the Poisson regression, negative binomial regression and their associated zero inflated and hurdle models were used to determine which offered the best fit to the count data.

**Results:**

Overall, 94.1% of the study participants did not have any schistosomiasis eggs out of 1345 people tested, resulting in a high zero inflation. The performance of the negative binomial regression models (hurdle negative binomial (HNB), zero inflated negative binomial (ZINB) and the standard negative binomial) were better than the Poisson-based regression models (Poisson, zero inflated Poisson, hurdle Poisson). The best models were the ZINB and HNB and their performances were indistinguishable according to information-based criteria test values.

**Conclusion:**

The zero-inflated negative binomial and hurdle negative binomial models were found to be the most satisfactory fit for modelling the over-dispersed zero inflated count data and are recommended for use in future statistical modelling analyses.

## Introduction

Schistosomiasis is a parasitic disease caused by trematode parasites of the genus *Schistosoma* [1]. It affects approximately 251.4 million people globally across 78 countries, with an estimated cases of 90% in Africa [2, 3]. The main causes of schistosomiasis are ***Schistosoma haematobium*** and ***S. mansoni*** [4]. The presence of S. haematobium results in genitourinary disease, while S. mansoni causes hepatobiliary schistosomiasis. Complications of schistosomiasis may include liver fibrosis, varices, and bladder carcinoma. Additionally, the infection is linked to anemia, deficiencies in nutrition, and stunted growth. It also has negative impacts on cognitive development, reducing physical activity, school performance, work capacity, and productivity [5]. Schistosomiasis results in high morbidity and socio-economic burden to affected individuals and communities, with risk factors including poor sanitation, prolonged exposure to infested freshwater bodies, and occupational hazards due to farming and fishing [6, 7].

In order to show the full burden of infection due to schistosomiasis, it is crucial to understand the epidemiology of the disease. The number of parasitic worms present in a group of people determines the transmission intensity of schistosomiasis, which is indirectly measured by the quantity of discharged eggs in stool or urine [8]. Appropriate statistical models can be used to study the epidemiology of infection. However, while using statistical techniques to analyze count data, methodological issues and the nature of the data must be taken into consideration [9].

Count data are common in health science research, especially when researchers investigate topics such as parasitic infections, COVID 19 among students, and the frequency of serious incidents in medical laboratories [10]. Count data can assume a probability distribution such as a Poisson. Count data, however, can also display more variability known as overdispersion (i.e., variance >mean) [11]. Often, a Poisson or negative binomial (NB) model cannot adequately account for the sample’s number of zeros and may result in distorted test statistics, goodness-of-fit and estimated standard errors [12]. Zero-inflated (ZI) and hurdle models are two models that are often used [9], to account for zero-inflated datasets. Further, hurdle models are an extension of the zero inflated (ZI) models. Hurdle models are also known as two part models [13], where the first part is a Bernoulli (for zero and non-zero counts) probability and the second part, which handles positive counts, is the zero-truncated Poisson (ZTP) or zero-truncated negative binomial (ZTNB) distribution [10]. Previous studies have demonstrated that if excessive zeros are not taken into consideration, both the zeros and nonzero counts would fit poorly [14].

The approach used to model zero counts is the primary difference between ZI and hurdle models. Zero observations in a ZI model [15] might come from either “sampling zeros” that are part of the underlying sampling distribution (Poisson, or negative binomial) and “structural zeros” that cannot score anything other than zero [16]. That is to say, the total number of zeros are divided into excess zeros and zeros generated by the distribution. Whereas the ZI models assume that both sources of zeros are involved, hurdle models assume only structural sources [10]. The typical Poisson or negative binomial distribution is used for the sampling of zeros, and it is presumed that they occur by chance [17]. This is the nature of the schistosomiasis count data, especially in low prevalence settings.

The purpose of this study is to assess the performance of the statistical models Poisson, NB and their related ZI and hurdle models in relation to different set of predictors and infection intensity for schistosomiasis to inform the effective analysis of data.

## Materials and methods

This is a secondary analysis of de-identified data collected within a larger epidemiological study on allergy and parasitic infections (“GLOFAL GHANA”) [18, 19]. The data constitute 1345 participants (635 male, 710 female)—within 12 communities in southern Ghana. These communities were grouped into two areas; rural and urban. The age range of participants was 4–21 years, distributed from nursery & kindergarten (considered as pre-school), primary and junior high school. The ages were grouped into three age groups with group one consisting of children aged 4–9, group two consisting of children aged 10–15 and group three consisting of children aged 16–21. Parents’ occupations were also grouped into the main occupational activities of farming and fishing, and others (including teaching, driving, masonry, trading, etc). Water sources used by communities are pipe borne water, tanker (treated), tanker (untreated), river/stream, well/borehole. The count data is available in S1 Appendix in the supporting information section below.

### Demographic covariates

The covariates considered for this analysis are the age group, sex, educational level, area (i.e. rural or urban), the occupation of parents, and sources of water usage. These covariates have been identified as predictors for schistosomiasis infection informed by literature [20–22]. These covariates, also taken as predictors, formed the regression matrix for the models used. The information about these covariates have been listed in Table 1.

**Table 1:** Data summary.

Definition	Information
Schistosomiasis egg counts	The number of eggs presents in an individual.
Age	The age of an individual; grouped into three categories (in years) which are <10, 10–15 and >15.
Sex	The sex of the individual, that is male or female.
Area	The area where individuals reside in. These are the rural and urban area.
Educational level	Each student’s class (i.e. educational level) in school. This is grouped into pre school, primary and Junior high.
Parent’s occupation	Occupation of the child’s parent, classified into farming, fishing and others.
Pipe borne	Water obtained from the tap.
Tanker (treated)	Treated water delivered by water tankers into the community.
Tanker (untreated)	Treated water delivered by water tankers into the community.
River/stream	Water from a nearby river/stream in the community.
Well/borehole	Water fetched from the well/borehole.

### Statistical methods

In a generalized linear model (GLM), the response variable is related to a linear combination of the regression variables through a non-linear link function [23],

$$\begin{array}{c} E\left(y|x\right)=g^{-1}(b_{0}+b_{1}x_{1}+b_{2}x_{2}+\ldots +b_{n}x_{n}+\epsilon ) \end{array}$$ (1)

Poisson regression is a type of GLM used to model discrete count data whereby the link function is exponential and *E*(*y*|*x*) ≔ λ represents the expected number of counts given X and is expressed as,

$$\begin{array}{c} \lambda ≔E\left(y|x\right)=\text{exp}(b_{0}+b_{1}x_{1}+b_{2}x_{2}+\ldots +\epsilon ) \end{array}$$ (2)

Inverting this the problem reduces to standard linear regression against the logarithm of the mean [24],

$$\begin{array}{cc} \text{log}\left(\lambda \right)=b_{0}+b_{1}x_{1}+b_{2}x_{2}+ & \ldots +\epsilon \end{array}$$ (3)

where *b*_0_, *b*_1_, …, *b*_*n*_ are the intercepts and slopes we fit to minimize the residual of the errors. The probability of observing the count *y*_*i*_ is Poisson distributed as per the following probability mass function:

$$\begin{array}{c} P\left(y_{i}|\lambda \right)=\frac{e^{-\lambda }\lambda^{y_{i}}}{y_{i}!},y_{i}=0,1,2,3,\ldots \end{array}$$ (4)

For more complex models, where the Poisson assumption does not hold, there may be more parameters such as overdispersion parameter *α*, or zero-truncation probability *π*, each of which can be fitted with a different link function and in a similar way [17].

For a given mean, the negative binomial distribution has a higher variance than the Poisson distribution [25]. Its probability mass function is expressed as

$$\begin{array}{c} P\left(y_{i},\lambda ,\alpha \right)=\frac{\Gamma (\alpha^{-1}+y_{i})}{\Gamma \left(\alpha^{-1}\right)\Gamma \left(y_{i}+1\right)}{\left(\frac{\alpha^{-1}}{\alpha^{-1}+\lambda }\right)}^{\alpha^{-1}}{\left(\frac{\lambda }{\lambda +\alpha^{-1}}\right)}^{y_{i}} \end{array}$$ (5)

The mean and variance of the negative binomial distribution are *E*(*y*) = λ, and *Var*(*y*) = λ(1 + *α*λ), where *α* is the dispersion parameter and if *α* = 0, negative binomial approaches the Poisson [24].

Zero-inflated models introduce extra probability mass to account for the excess zeros in the outcome. This results in a two-state mixture distribution with a PMF defined by the ZIP model;

$$\begin{array}{c} P\left(y_{i}|\lambda \right)=\{\begin{array}{cc} \pi +\left(1-\pi \right)e^{-\lambda }\; & \text{if}\;y_{i}=0\\ \left(1-\pi \right)\frac{e^{-\lambda }{\left(\lambda \right)}^{y_{i}}}{y_{i}!}\; & \text{if}\;y_{i}=1,2,3,\ldots \end{array} \end{array}$$ (6)

In a case where overdispersion and excess zeros occur in data, recommending the search for an alternate model to the ZIP model. The zero inflated negative binomial (ZINB) is given by the equation,

$$\begin{array}{c} P\left(y_{i}|\lambda ,\alpha \right)=\{\begin{array}{cc} \pi +\left(1-\pi \right){\left(1+\alpha \lambda \right)}^{-\alpha^{-1}}\; & \text{if}\;y_{i}=0\\ \left(1-\pi \right)\frac{\Gamma (y_{i}+\frac{1}{\alpha }){\left(\alpha \lambda \right)}^{y_{i}}}{y_{i}!(\frac{1}{\alpha })1+\alpha \lambda^{y_{i}+\frac{1}{\alpha }}}\; & \text{if}\;y_{i}=1,2,3,\ldots \end{array} \end{array}$$ (7)

where *y*_*i*_ is the observed counts and *π* is the probability of non-infection. Eq (7) models two processes, where the first process generates the excess zeros, with the probability *π*, and the second one uses the negative binomial to generate counts (including zeros).

In contrast, the hurdle models also have two components which are the zero count and the positive counts for both the Poisson and negative binomial model [9]. The first component handles the zero count and the other part deals with the positive counts which is referred to as the zero truncated Poisson or NB [17]. In Eqs (8) and (9) below, (1 − *π*) is the likelihood of overcoming the “hurdle” and generating a non-zero count [26]. The hurdle Poisson model is expressed as

$$\begin{array}{c} P\left(y_{i}|\lambda \right)=\{\begin{array}{cc} \pi \; & \text{if}\;y_{i}=0\\ \left(1-\pi \right)\frac{e^{-\lambda }\lambda^{y_{i}}}{y_{i}!}\; & \text{if}\;y_{i}=1,2,3,\ldots \end{array} \end{array}$$ (8)

For the negative binomial it would look as follows:

$$\begin{array}{c} P\left(y_{i}|\lambda ,\alpha \right)=\{\begin{array}{cc} \pi \; & \text{if}\;y_{i}=0\\ \left(1-\pi \right)\frac{\pi }{1-{\left(\frac{\alpha }{\lambda +\alpha }\right)}^{\alpha }}\frac{\Gamma (y+\alpha )}{\Gamma (\alpha )y!}{\left[\frac{\lambda }{\lambda +\alpha }\right]}^{y}{\left[\frac{\alpha }{\lambda +\alpha }\right]}^{\alpha }\; & \text{if}\;y_{i}=1,2,3,\ldots \end{array} \end{array}$$ (9)

where *y*_*i*_ is the observed counts taking values *y* = 0, 1, 2, ‥ and *π* is the probability of non-infection. Also, the second equation in the piecewise function of Eqs (8) and (9) are the zero-truncated Poisson and zero-truncated negative binomial distribution respectively.

The parameters of each model under different socio-demographic predictors were estimated by using maximum likelihood estimation (MLE) to simulate the model. The binary component, which determines if an observed count is zero (negative infection) or non-zero (positive infection), of the HNB model uses a logit binary link [10],

$$\begin{array}{c} logit\;(\rho )=\gamma_{0}+\gamma X \end{array}$$ (10)

and the schistosomiasis positive counts as a dependent variable for our proposed regression model is

$$\begin{array}{c} \text{log}\left(\mu \right)=\beta_{0}+\beta X \end{array}$$ (11)

where *X* is the regression variable matrix, *γ*_0_, *γ*, *β*_0_ and *β* are the vectors of parameters.

### Models comparison

Several tests have been used for comparing models. An example is the Vuoung test [27] for non-nested models, such as ZINB and HNB, in several applications. Alternatives include the Akaike, Bayesian information-theoretic criteria and an approach that embeds the alternative models in an artificial compound model [26, 28]. We used the Akaike and Bayesian information criteria to understand the model performances against each other and discriminate against other tests. The Akaike Information Criteria (AIC) is likely to perform well when heterogeneity is small. However, when heterogeneity is large, which may result in overfitting, the Bayesian information criterion (BIC) will often perform better because of the stronger penalty provided [29]. By adding a penalty term for the number of parameters in the model, the BIC solves this issue. The AIC and BIC were used to test the goodness-of-fit of the models [30]. The mathematical formulation of the AIC/BIC with their basic properties is presented in Jiawei’s paper [31]. Better model fit is indicated by a lower AIC or BIC value indicating the data is more likely to have derived from the distribution in question. Both tests are based on the MLE method. The formula for the AIC is given as follows:

$$\begin{array}{c} \text{AIC}=2k-2\;\text{ln}(\hat{L}) \end{array}$$

where $\hat{L}$ represents the log-likelihood of the data under the model, and *k* is the number of model parameters that is the number of variables and the intercept in the model [32]. The BIC is formally defined as

$$\begin{array}{c} \text{BIC}=k\;\text{ln}\left(n\right)-2\;\text{ln}\left(\hat{L}\right) \end{array}$$

where $\hat{L}$ is the maximized value of the likelihood function, *n* is the sample size and *k* is the number of parameters estimated by the model.

Models compared are Poisson, NB, ZIP, ZINB, HP and HNB and if the discrepancy in the AIC or BIC values between competing models is less than 2.5, they cannot be distinguished, whereas a difference exceeding 10 (>10) indicates substantial evidence in favour of the model with the lowest criterion [9]. However, if the difference between two models lies in the range 2.5–6 then the preferred model is that with the smallest value if the sample size *n* >256. In the same light, if the difference between the models is in the range 6–9 then the preferred the model is the one with the lowest value if also *n* >64 [30].

The statsmodels, pandas and matplotlib libraries in python version 3.11.8 and political science computational laboratory (PSCL) package in the statistical software R version 4.3.1 were used for model fitting [33] and plotting figures.

### Measures of association

Modelling was done using the most significant variables from the cross-tabulation with the chi-square test. However, the output results of each model include estimated *p*-values representing the association of a predictor, in relation with other predictors, to the dependent variable (schistosomiasis counts). So, we tabulated infection status paired with each of the chosen predictor individually, aiming to investigate association separately with the infection status, having a binary outcome (positive or negative).

### Ethics statement

Not applicable. This is a secondary analysis of previously collected data. All data was de-identified prior to receipt from the original study investigators.

## Results

### Descriptive statistics

**Fig 1:**
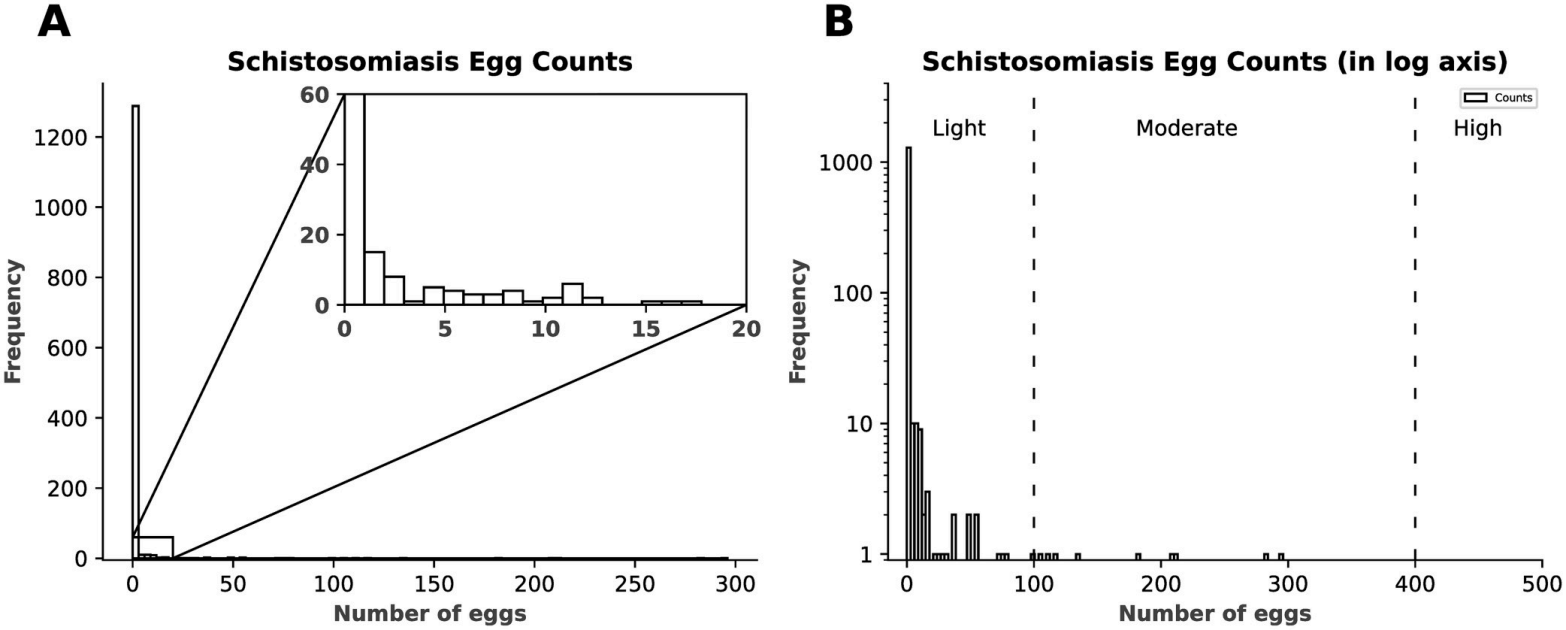
Schistosomiasis egg counts. A: Histogram showing the distribution of egg counts for schistosomiasis; with sample size 1345. B: Histogram showing the distribution of egg counts for schistosomiasis in log axis. The divided regions; light (1–99 epg), moderate (100–400 epg) and high (>400 epg), equate the infection intensity count categories.

A large percentage (94.1%) of the count data were zeros (non-infected individuals), with the remainder being positive counts of infected individuals. The median age of participants was 11 years. The mean of schistosomiasis egg count, excluding the zeros, was 33.4 egg per gram, with a standard deviation of 61.8 and an over-dispersion parameter *α* = 2.1. The distribution of the count data is shown in Fig 1A.

### Grouping by infection intensity

Light, moderate, and high infection intensity categories are defined as counts between 1–99, 100–399, ≥400 epg respectively [34]. From the histogram of these counts given in Fig 1B, although the distribution is positively skewed there are a few gaps between counts after the 100 mark on the horizontal axis (or light intensity range). Due to this we took the low intensity count of the data with zeros (n = 1336, mean = 0.8 epg, variance = 33.3) and the “all intensity” count data (i.e. all sample) to evaluate the model performances. Upon excluding the moderate (n = 9, mean = 183.6 epg, variance = 5275) with no value in the high intensity range, the mean and variance of the low intensity sample became smaller than that of the actual mean (2 epg, including the zeros) and variance (286.7) of the count data but still resulted in over-dispersion (variance >mean) with the overdispersion parameter *α* = 74.0. We assessed how the model performed by neglecting moderate and high intensity values (>100 epg) with the same predictors and made a comparison between both levels of intensity. The results of this assessment, presented in Table 2, show a significant difference in both information criteria values between the two levels of intensity. An approximated difference, between using low intensity and all intensity values, of 200 was observed, against all levels of intensity, implying a better performance by the low intensity values compared to using all values.

**Table 2:** Model’s AIC and BIC fit summary statistics.

Intensity	Predictors	Test	Poisson	NB	ZIP	ZINB	HP	HNB
All	Five	AIC	14704	3464	4828	1206	4828	**1203**
		BIC	14751	3511	4922	1305	4922	**1302**
	Ten	AIC	14128	3216	4405	1209	4405	**1207**
		BIC	14201	3288	4550	1360	4551	**1358**
Low	Five	AIC	6533	2645	2070	**1024**	2069	**1024**
		BIC	6580	2692	2153	**1123**	2163	**1123**
	Ten	AIC	6207	2494	1937	**1026**	1938	1033
		BIC	6279	2567	2083	**1177**	2083	1183

The lowest AIC and BIC values for each scenario is marked in bold. From the reported AICs and BICs, the ZINB model is comparable to the HNB model.

**Fig 2:**
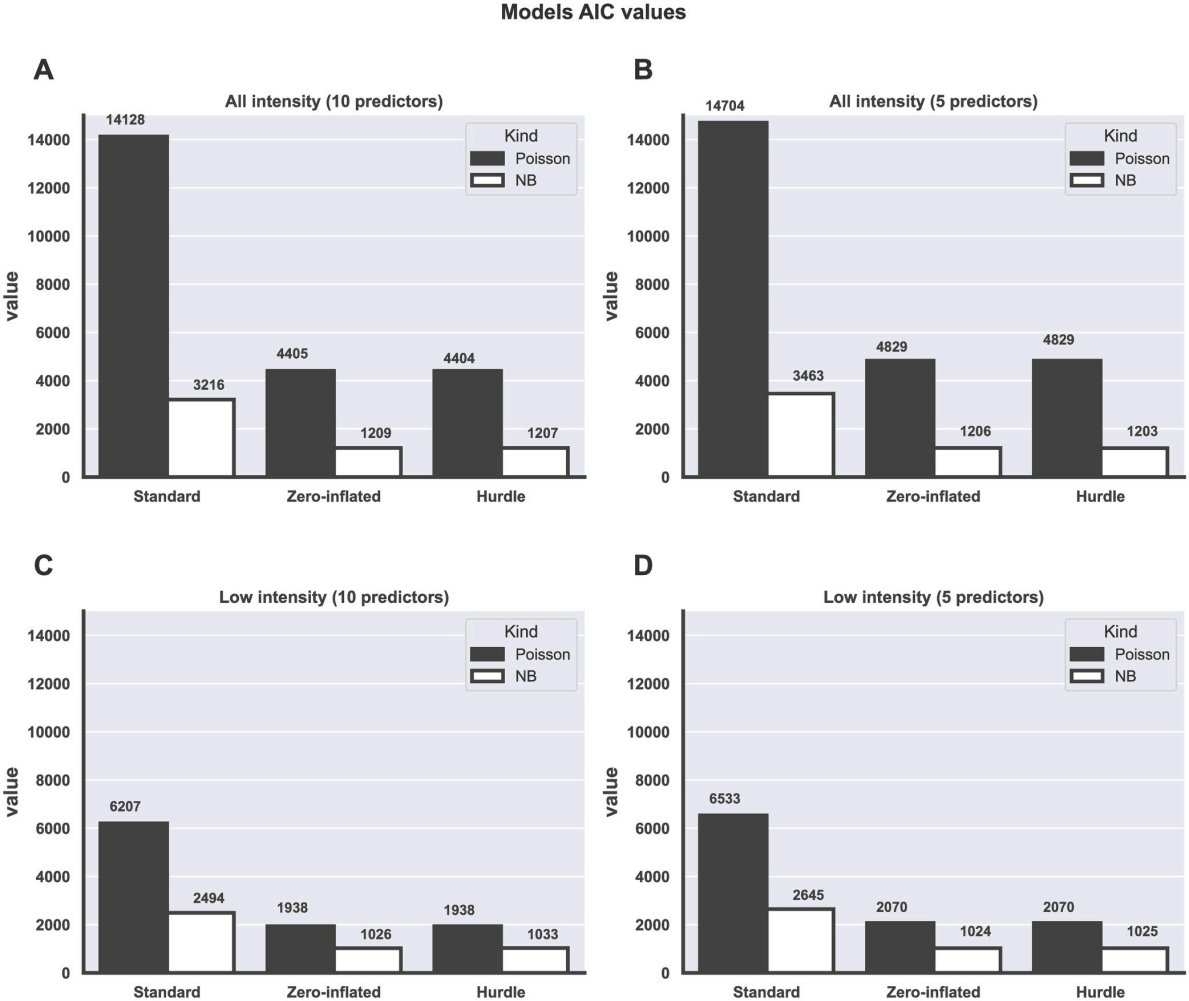
Comparison of the AIC values of models used. Comparison of the AIC values between Poisson and NB-based regression models (the standard, ZI and hurdle). This graph displays the AIC values for each model under different scenarios; A. shows all intensity (i.e. all sample (n = 1345)) simulated with 10 predictors. B. presents all sample size simulated with 5 predictors. C shows the low intensity samples (n = 1336) simulated with 10 predictors. D shows low intensity samples simulated with 5 predictors. For each sub-figure, the AIC value for the negative binomial-based models is much greater than its corresponding Poisson-based model.

From Table 2, it is observed that the difference between the AIC and BIC values of the ZINB and HNB model for using five predictors scenario is ≤3. Hence their performance is indistinguishable (AIC = 1206, BIC = 1305 for ZINB; AIC = 1203, BIC = 1302 for HNB). Also, a difference ≤3 between ZINB and HNB was observed using the ten predictors (all intensity) and the implication of this result is that, the ZINB model did not outperformed the HNB model under this scenario (AIC = 1209, BIC = 1360 for ZINB; AIC = 1207, BIC = 1358 for HNB). Overall, the NB and its associated models (NB, ZINB, HP) performed better than the Poisson-based models (Poisson, ZIP, HP) as shown in Fig 2 where a greater difference between the NB-based model’s AIC values and the Poisson-based models.

### Using different set of predictors

Different sets of variables were used to determine the performance of the model. The first five predictor variables were age groups, sex, educational level, area and parent’s occupation. The next ten predictor variables used were the first five in addition to their sources of water namely pipe-borne water, treated water from water tanker, untreated water from water tankers, river/stream and well/borehole. Table 2 shows their respective performance on the model. As a result, the AIC and BIC values for using five predictors are greater than that of the ten predictors for each model and scenario except for the HNB model results. For the HNB test values, using all intensity, the difference in the five predictor’s AIC value (1203) and ten predictors AIC value (1207) is 4. Hence, the best fit is in favour of both using both predictors. Similarly, the difference in their BIC values is 56, favouring the use of the 10 predictors (which has the lowest BIC value). However, the *p*-value of the likelihood ratio test between using the five and ten predictor for the HNB is not <0.05, concluding that compared to the 5 predictors, the 10 predictors did not give a significant fit improvement.

### Interpreting model fitting

**Fig 3:**
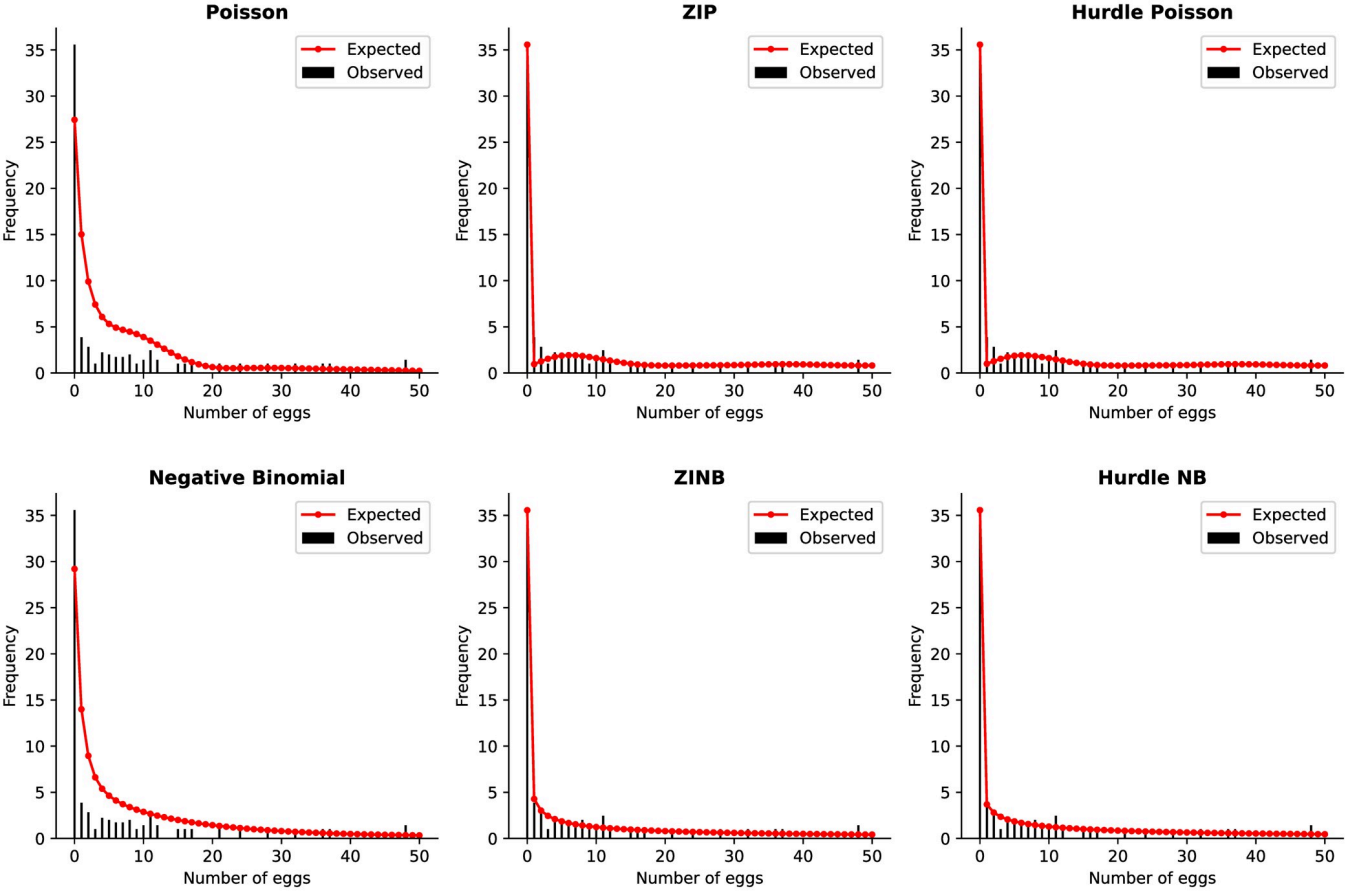
Each model’s predictions overlaid on the observed bar counts. The hurdle Poisson and hurdle negative binomial both captured 1265 zeros, which is equal to the observed zero count. Additionally, zero captured for zero inflated Poisson (ZIP) is 1265, zero inflated negative binomial (ZINB) is 1264 but the Poisson and NB model underestimated the zeros (513 and 412 zeros respectively) and overestimated the positive counts. The ZIP and HP expected values were lower than the observed for the first 10 counts whilst ZINB and HNB expected values were almost equal to the observed with a difference by a small margin.

**Fig 4:**
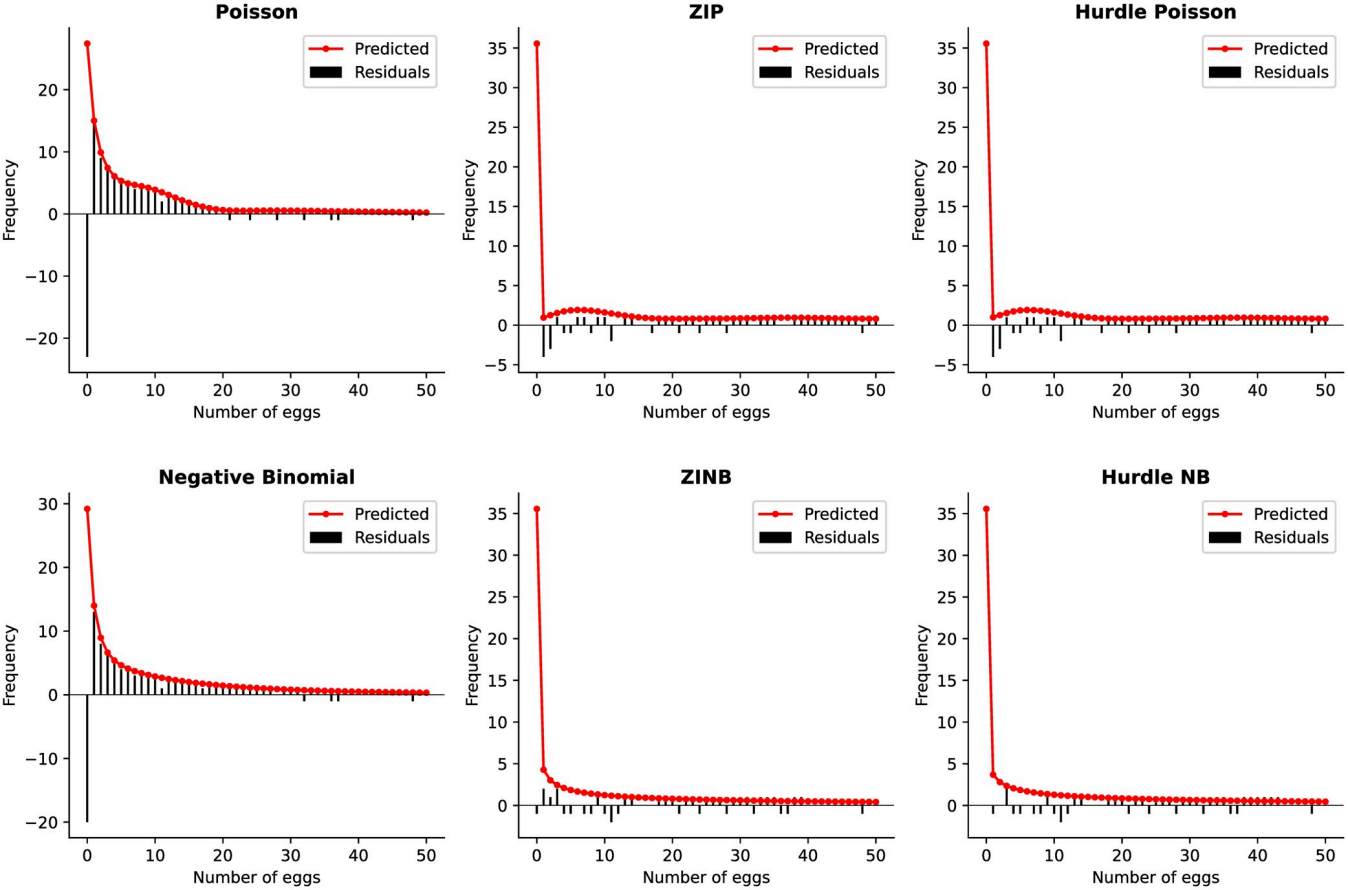
Alternate plot showing the low and high values of each model’s prediction. Here, the $\sqrt{Frequency}=\sqrt{Expected}-\sqrt{Observed}$ but not $\sqrt{Expected-Observed}$. The standard Poisson and negative regression models did not account for all the zeros observed; with zeros difference of 513 and 412 for Poisson and negative binomial from the number of observed zeros (low prediction). Moreover, the zero-inflated and hurdle models accounted for all zeros. However, we observe an overestimation for the positive counts for the standard Poisson and both underestimation and overestimation for the other models.

To evaluate the fit of regression models, individuals regularly examine the deviations of observations from predicted values using many approaches. One way to achieve this is when the observed frequencies are plotted against or overlaid on the predicted/expected values. Rootograms, as proposed by Kleiber and Zeileis [35] is a new way to assess count models, such that it presents the square roots of the predicted values as a continuous curve overlaid on that of the bars of observed counts. Fig 3 simply shows the rootogram for the first 50 number of eggs’ frequencies. Further, Fig 4 shows the differences between observed and expected counts, with bars hanging above and below from the zero line to highlight overestimated and underestimated values respectively, providing information about the model residuals rather than the fitted values shown in Fig 3.

Figs 3 and 4 highlight that the Poisson model underestimated the zeros (752 zeros captured) and overestimated the positive counts, with a difference of 211 for positive count 1, 90 for count 2, 54 for count 3 and so on; whilst the negative binomial model predicted less zeros (853 captured; overestimating) and positive counts which were overestimated (positive count 1: overestimated by 183; 2 by 72; 3 by 43, …). Due to overdispersion and zero-inflation, the standard Poisson and negative binomial model were too high for low values. The zero-inflated and hurdle model’s zero prediction was equal to the number of zeros observed (1265). From Fig 3, without the ZINB and HNB models, the ZIP and HP was a bit high for low values as the counts moves away from zero but gave a better approximation to the observed frequencies, as it gets larger. S1 Fig presents the rectangles/bars of the observed frequencies plotted against the predicted ones for first 15 egg counts and in log axis.

### ZINB and HNB regression model

The regression output results for the zero-inflated negative binomial and hurdle model parameter estimates where all sample size with ten predictors was used to simulate the models are shown in Table 3. From the results shown in Table 3, it can be seen that two variables (area, pipe borne, water by tankers (untreated) and well/borehole) of the negave variables (age groups <10 and 10–15, area, pre-school (educational level) and untreated water from tankers) have a significant effect as their p-values (Pr(>|Z |)) <0.05 for the ZINB and HNB model respectively. Also, three variables—area, pipe-borne water and water from well/borehole were significant with the positive counts with p-values, 0.017, 0.015 and 0.009 respectively for the ZINB model. Four variables—area, pipe-borne water, water from tankers (untreated) and well/borehole were significant with the positive counts with the p-values 0.009, 0.021, 0.005 and 0.002 respectively. Their respective standard errors too are attached.

**Table 3:** Zero hurdle model coefficients (binomial with logit link) and count model coefficients (truncated negbin with log link) with 95% CI.

	ZINB				HNB			
Parameter	Est.	Std. Error	z value	Pr(> |*z*|)	Est.	Std. Error	z value	Pr(> |*z*|)
**Negative Binomial Model (Log Link)**					**Negative Binomial Model (Log Link)**			
**Intercept**	1.20	2.35	0.51	0.6112	3.20	2.21	1.45	0.1482
**Age (in years)**								
10–15	0.46	0.84	0.55	0.5818	0.63	0.62	1.02	0.3082
>15	-0.57	2.20	-0.26	0.7936	1.80	1.96	0.92	0.3591
**Sex**	0.01	0.66	0.02	0.9856	0.30	0.57	0.53	0.5949
**Area**	-2.32	0.98	-2.38	0.0174*	-2.29	0.88	-2.60	0.0093**
**Educational level**								
PreSchool	-0.44	2.38	-0.19	0.8523	-1.51	2.17	-0.70	0.4856
Primary	-2.84	2.18	-1.30	0.1929	-2.91	2.08	-1.40	0.1608
**Parent’s occupation**								
Fishing	0.32	1.05	0.30	0.7627	-1.58	0.81	-1.93	0.0531.
Others	1.34	0.79	1.70	0.0898*	-0.35	0.72	-0.48	0.6295
**Water sources**								
Pipe borne	3.52	1.45	2.43	0.0153*	2.79	1.21	2.31	0.0211*
Tanker (treated)	3.60	4.66	0.77	0.4393	0.91	1.92	0.476	0.6339
Tanker (untreated)	1.52	0.87	1.76	0.0787*	2.44	0.88	2.78	0.0054**
River/Stream	0.45	1.05	0.43	0.6663	-0.35	0.92	-0.38	0.7075
Well/borehole	2.22	0.85	2.61	0.0092**	2.92	0.94	3.11	0.0019**
Log(theta)	-3.30	0.19	-17.76	<0.0001***	-1.48	0.50	-2.96	0.0031**
**Zero-inflation (Binomial)**					**Zero-inflation (Binomial)**			
(Intercept)	-15.37	3685.52	-0.01	0.9967	-5.55	1.20	-4.64	<0.0001***
**Age (in years)**								
>15	-4.73	2.25	-2.10	0.0358*	1.98	1.14	1.73	0.0829.
10–15	-3.63	1.02	-3.57	0.0003***	1.86	0.42	4.37	<0.0001***
**Sex**	-1.02	0.68	-1.50	0.1343	0.16	0.24	0.66	0.5112
**Area**	1.97	1.37	1.44	0.1494	-1.23	0.52	-2.38	0.0171*
**Educational level**								
PreSchool	-2.68	1.67	-1.61	0.1084	2.60	1.15	2.25	0.0242*
Primary	-1.73	1.32	-1.31	0.1904	0.93	1.04	0.89	0.3714
**Parent’s occupation**								
Fishing	19.96	3685.52	0.01	0.9957	0.22	0.39	0.56	0.5779
Others	19.95	3685.52	0.01	0.9957	0.24	0.32	0.73	0.4628
**Pipe borne**	0.80	2.27	0.35	0.7246	0.39	0.67	0.58	0.5628
**Tanker (treated)**	18.67	3685.52	0.01	0.9959	-0.88	1.14	-0.78	0.4379
**Tanker (untreated)**	-1.18	2.09	-0.56	0.5739	0.80	0.41	1.96	0.0495*
**River/stream**	-0.53	3.44	-0.15	0.8772	0.54	0.56	0.97	0.3312
**Well/borehole**	-0.07	2.15	-0.03	0.9727	0.54	0.45	1.22	0.2241

Parameter estimates for the zero inflated negative binomial and hurdle negative binomial results.

**refers to significant difference at p<.01.

***refers to significant difference at p<.001.

### Modelling and interpreting main effects

Table 4 shows the odds ratios (ORs) obtained from the logistic portion of the HNB model. Some confidence intervals for the ORs were observed to be wide. From Table 4, it is noted that people who live in urban areas have a lower risk of getting schistosomiasis infection as compared to people who live in rural areas. We can assess that children within the ages 10–15 (OR = 1.88, 95% CI: (0.56, 6.32)) have a higher risk of getting infected as compared to children whose age are greater than 15. Also, children who use untreated water delivered by water tankers (OR = 2.33, 95% CI: (1.00, 4.97)) have a greater risk of harboring schistosomiasis egg counts than those who use water from other sources.

**Table 4:** Zero hurdle model coefficients (binomial with logit link) and count model coefficients (truncated negbin with log link) with 95% CI.

Parameter	Infection probability OR (95% CI)	Infection intensity OR (95% CI)
**Age (in years)**		
<10	—	—
10–15	1.88 (0.56, 6.32)	6.39 (2.78, 14.7)
>15	6.04 (0.13, 283)	7.21 (0.77, 67.2)
**Sex**		
Female	—	—
Male	1.35 (0.45, 4.10)	1.17 (0.73, 1.87)
**Area**		
Rural	—	—
Urban	0.10 (0.02, 0.57)	0.29 (0.11, 0.80)
**Educational level**		
JuniorHigh	—	—
PreSchool	0.22 (0.00, 15.5)	13.5 (1.40, 130)
Primary	0.05 (0.00, 3.18)	2.54 (0.33, 19.5)
**Parent’s occupation**		
Farming	—	—
Fishing	0.21 (0.04, 1.02)	1.24 (0.58, 2.67)
Others	0.71 (0.17, 2.90)	1.27 (0.67, 2.38)
**Pipe borne**		
FALSE	—	—
TRUE	16.3 (1.52, 174)	1.47 (0.40, 5.48)
**Tanker (treated)**		
FALSE	—	—
TRUE	0.41 (0.04, 3.84)	0.41 (0.04, 3.84)
**Tanker (untreated)**		
FALSE	—	—
TRUE	11.5 (2.06, 64.2)	2.23 (1.00, 4.97)
**River/stream**		
FALSE	—	—
TRUE	0.71 (0.12, 4.31)	1.72 (0.58, 5.15)
**Well/borehole**		
FALSE	—	—
TRUE	18.5 (2.95, 116)	1.72 (0.72, 4.11)

Odd ratio from the hurdle negative binomial results.

## Discussion

Schistosomiasis, an ailment caused by blood flukes of the Schistosoma genus, is a waterborne helminthic disease [36]. It predominantly affects underprivileged rural communities residing in tropical and subtropical areas, where access to safe drinking water and proper sanitation is limited. Infection occurs when individuals come into contact with infested natural freshwater bodies while engaging in activities such as fishing, agriculture, laundry, or swimming [37].

For the analysis of schistosomiasis egg count data, six distinct models applying either the Poisson or negative binomial distribution were taken into account. Various scenarios were considered to simulate the model, however, the focus of the discussions will be on the results when ten predictors and all the sample data was used. The count data was overdispersed and zero-inflated. The standard Poisson and NB performed poorly when fitted to the count data since they both recorded a higher information-theoretic criteria’s value in Table 2 compared to the zero-inflated (ZI) and hurdle models. Further, the ZI and hurdle models were fitted to the count data and by the AIC and BIC fit statistics with the model’s predictions shown in Fig 3 indicated that the ZINB (AIC = 1209, BIC = 1360) and HNB (AIC = 1207, BIC = 1358) model best fitted the data. Table 5 presents each model’s number of zeros captured when five and ten predictors were used against all the sample data and both the standard Poisson (717 zeros captured) and NB (801 zeros captured) failed to capture the number of observed zeros (1265 zeros) with a difference of 548 and 464 respectively when five predictors were used. Additionally, by introducing extra predictors, the Poisson (752 zeros captured) and NB (853 zeros captured) did not the number of observed zeros with a difference of 513 and 412 respectively. On the other hand, the ZI and hurdle models effectively accounted for all 1265 observed zeros when five predictors were employed. By incorporating additional predictors, all ZI and hurdle models successfully captured all zeros, except for the ZINB model which captured 1264 zeros, resulting in a difference of 1. This implies that there is no necessity to introduce additional parameters for capturing the number of observed zeros in the ZI and hurdle models. These findings emphasize the advantages of using ZI and hurdle models over the standard Poisson and NB models for analyzing zero-inflated count data. Moreover, the likelihood ratio test comparing the use of five and ten predictors for the ZINB model (p-value = 0.0711) and HNB model (p-value = 0.0969) does not show significance.

**Table 5:** Model’s zero capturing.

Predictors	Observed	Poisson	NB	ZIP	ZINB	HP	HNB
Five	1265	717	801	1265	1265	1265	1265
Ten	1265	752	853	1265	1264	1265	1265

The number of zero predicted by each model. The predictions by the standard Poisson and NB varied for each predictor used and their values were low. The predictions of all the zero-inflated and hurdle models matched the number of observed zeros under both predictors except for ZINB using 10 predictors with a difference of 1.

A review study [36] showed that the southern regions in Ghana have a mean schistosomiasis prevalence of 25.8% with a range of 3.3% to 83.9%. In this study, the infection prevalence among participant was 5.9%. We assessed the infection status and intensity in relation to factors such as age, sex, educational level, area of residence, parent’s occupation and their water sources. We found out that the factors linked to schistosomiasis infection include age (10–15 years), area of residence, pre-school education level, and the use of untreated water distributed by tankers. Furthermore, the factors associated with the intensity of schistosomiasis infection (positive counts) include place of residence and water sources such as untreated water from tankers, piped water, and well/borehole water. This confirms that high risk of infection are associated with specific areas and water sources [38–40]. Some research [41, 42] have shown that infection among children is associated with parent’s occupation but the occupation of parents did not show any significant influence on the involvement in schistosomiasis egg counts in this study. The implementation of control strategies is necessary to monitor the water sources, educate adults in rural areas on schistosomiasis infection, and guide parents in preventing their preschool children from accessing contaminated water. However, the results imply that safe drinking and well-treated water must be made available in endemic areas. An integrated approach to control that include sanitation improvement, health education, and less human-water interaction is required for success [43].

## Conclusion

The zero-inflated negative binomial (ZINB) and hurdle negative binomial (HNB) models, which are frequently employed to model zero-inflated count data with under-dispersion and over-dispersion, provided the most satisfactory fit among all the models used and performed the best. These models (ZINB and HNB) were used to identify key factors affecting the quantity of schistosomiasis egg counts in children. Factors such as age (10–15 years), residence in rural areas, pre-school educational level, reliance on untreated water sources like well/borehole water, untreated waters from tankers, and piped water were found to be associated with the numbers of positive egg counts. It was observed that the hurdle negative binomial outperformed the zero-inflated negative binomial model when ten set of predictors were used and this is based on the information-theoretic criteria used. Moreover, all zero-inflated models accommodated the excess zeros, recommending it for modelling count data with excess zeros. Extending the modelling approaches to perform geospatial analysis to predict areas of high transmission in order to inform control strategies is recommended.

## Supporting information

S1 Fig A bar relationship between observed and model’s expected frequencies (in log axis). (TIF)

S1 Appendix Data and code availability. The data, R and python codes for model comparison and analysis can be found at https://github.com/kojonketia/Using-hurdle-models-to-analyze-schistosomiasis-count-data.
(DOCX)
