# Supplementary figures and images for: Using zero-inflated and hurdle regression models to analyze schistosomiasis data of school children in the southern areas of Ghana
Source: PLoS One. 2024 Jul 12;19(7):e0304681. doi: 10.1371/journal.pone.0304681 (PMC11244785; doi:10.1371/journal.pone.0304681)

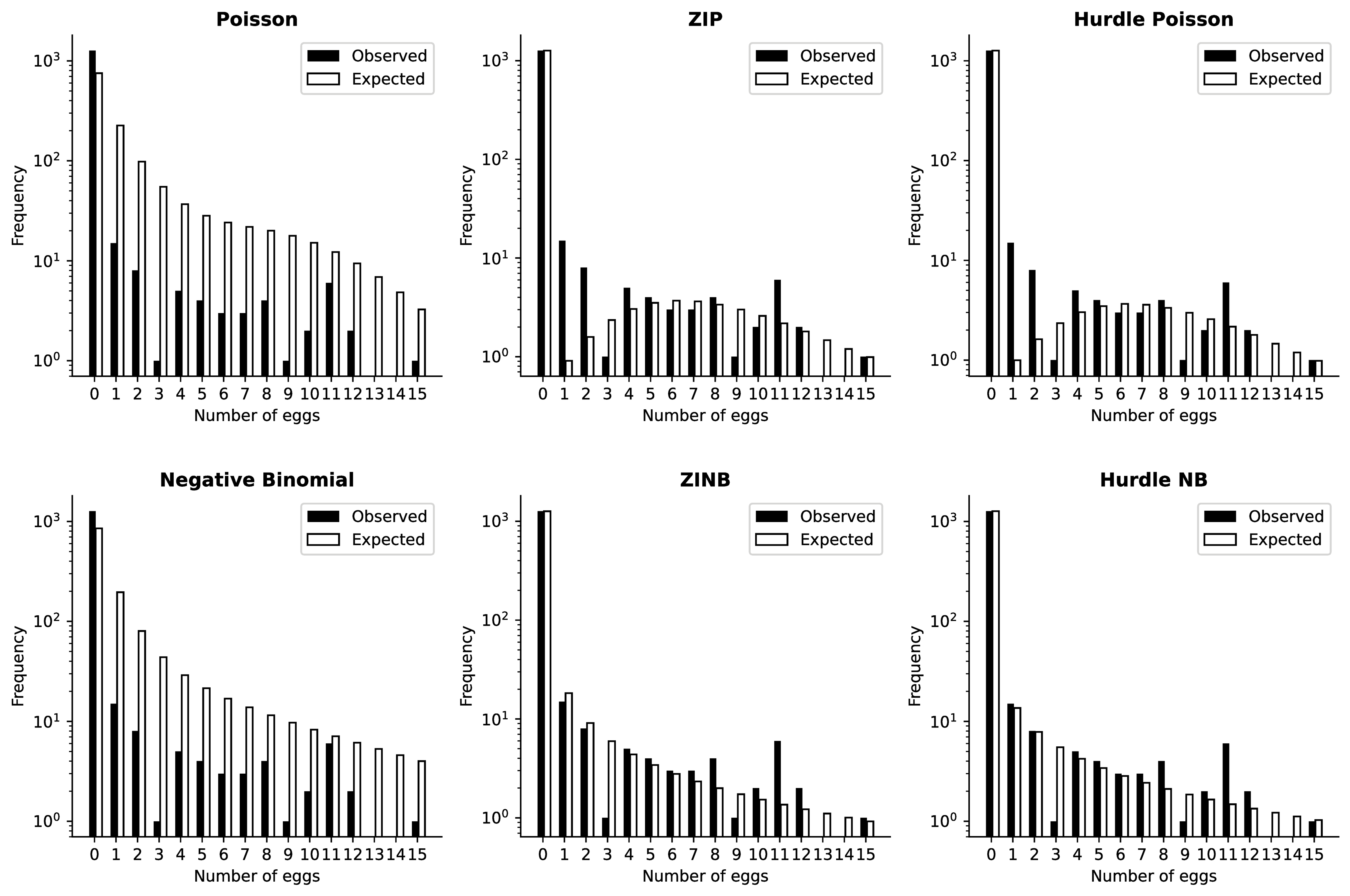

Supplement: S1 Fig — (TIF) [file pone.0304681.s001.tif]
