# Supplementary material for: Using zero-inflated and hurdle regression models to analyze schistosomiasis data of school children in the southern areas of Ghana
Source: PLoS One. 2024 Jul 12;19(7):e0304681. doi: 10.1371/journal.pone.0304681 (PMC11244785; doi:10.1371/journal.pone.0304681)
Supplement: S1 Appendix — The data, R and python codes for model comparison and analysis can be found at https://github.com/kojonketia/Using-hurdle-models-to-analyze-schistosomiasis-count-data. (DOCX) [file pone.0304681.s002.docx]

**S1 Appendix. Data and code availability.** The data, R and python codes for model comparison and analysis can be found at <https://github.com/kojonketia/Using-hurdle-models-to-analyze-schistosomiasis-count-data>
